# Thrombose de la veine porte révélant un syndrome de Cushing du sujet âgé

**DOI:** 10.11604/pamj.2014.18.186.4925

**Published:** 2014-07-04

**Authors:** Wafa Chebbi, Olfa Berriche

**Affiliations:** 1 Service de Médecine Interne, CHU Taher Sfar Mahdia, 5100 Mahdia, Tunisie

**Keywords:** Thrombose, veine porte, syndrome de Cushing, sujet âgé, thrombosis, portal vein, Cushing syndrome, elderly

## Image en médecine

La fréquence des thromboses veineuses profondes au cours du syndrome de Cushing varie de 7 à 25%. Elles sont secondaires à des mécanismes multiples: élévation des taux des facteurs VIII, IX et de Von Willebrand, une réduction du TCA et une augmentation du TP. Nous rapportons l'observation d'une thrombose de la veine porte dont l'exploration étiologique a conduit au diagnostic du syndrome de Cushing. Il s'agissait d'une patiente âgée de 66 ans, sans antécédents pathologiques, hospitalisée pour une douleur intense et brutale de l'hypocondre droit. L'examen trouvait une obésité facio-tronculaire, une tension artérielle à 150/90 mmHg, des vergetures larges et pourpres au niveau du ventre et une sensibilité de l'hypochondre droit. Le scanner abdominal objectivait une thrombose de la branche portale gauche et un adénome surrénalien droit de 2 cm de grand axe, de densité spontanée estimée à 0,4 UH et un Wash out supérieur à 50%. Le bilan hormonal montrait un taux normal d'acide vanylmandélique urinaire, une élévation du cortisol libre urinaire à 233 µg/24h avec une cortisolémie sous freinage minute à 76 ng/ml. Le bilan étiologique de la thrombose de la veine porte était négatif (hémogramme normal, absence de lésion tumorale hépatique et de foyer infectieux intra-abdominal, taux normaux de protéine C, d'anti-thrombine, de protéine S et de résistance à la protéine C activée, anticorps antiphospholipides négatifs). La patiente était mise sous anticoagulant et confiée à la chirurgie. La recherche des signes cliniques et biologiques du syndrome de Cushing doit figurer dans le bilan étiologique d'une thrombose veineuse portale sans cause évidente.

**Figure 1:**
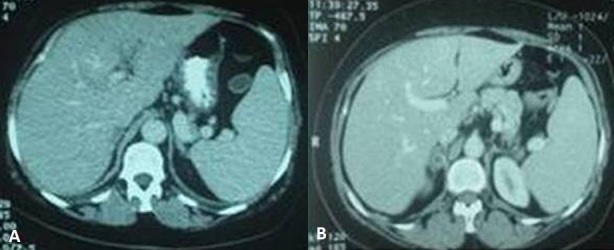
TDM abdominale en coupes axiales. A): thrombose de la branche gauche de la veine porte; B): adénome surrénalien droit

